# Independent mechanisms of T cell-suppression by subpopulations of myeloid-derived suppressor cells (MDSC) in tumor-bearing hosts

**DOI:** 10.1186/2051-1426-1-S1-P193

**Published:** 2013-11-07

**Authors:** Patrick L Raber, Paul Thevenot, Rosa Sierra, Dorota Wyczechowska, Maria E Ramirez, Augusto Ochoa, Cruz Velasco, Paulo C Rodriguez

**Affiliations:** 1 Department of Microbiology, Immunology and Parasitology, Louisiana State University Health Sciences Center, New Orleans, LA, USA; 2 Stanley S. Scott Cancer Center, Louisiana State University Health Sciences Center, New Orleans, LA, USA; 3 Department of Pediatrics, Louisiana State University Health Sciences Center, New Orleans, LA, USA

## 

Suppression of effector immune responses by the tumor microenvironment remains a major obstacle in the development of new therapies for cancer. The accumulation of myeloid-derived suppressor cells (MDSC) in tumor-bearing hosts is a hallmark of malignancy-associated inflammation and a major mediator for the induction of T cell suppression by tumors. MDSC can be divided phenotypically into granulocytic (G-MDSC) and monocytic (Mo-MDSC) subgroups. Previous studies have identified several mechanisms of how MDSC impair T cell function; however, the specific role of these pathways in the inhibitory activity of MDSC subpopulations remains unclear. Therefore, we aimed to determine the effector mechanisms by which subsets of tumor-infiltrating MDSC block T cell function. Our results showed that G-MDSC displayed a higher ability to impair proliferation and expression of effector molecules in activated T cells, as compared to Mo-MDSC. This effect was validated using different transplantable tumor models. Interestingly, both MDSC subgroups inhibited T cells through nitric oxide (NO)-related pathways, but expressed different effector inhibitory mechanisms. Specifically, G-MDSC impaired T cells through the production of peroxynitrites (PNT), while Mo-MDSC suppressed by the release of NO. The production of PNT in G-MDSC depended on the expression of NADPH oxidase subunit gp91phox and endothelial NO synthase (eNOS), while inducible NO synthase (iNOS) mediated the generation of NO in Mo-MDSC. Deletion of eNOS and gp91phox or scavenging of PNT blocked the suppressive function of G-MDSC and induced anti-tumoral effects, without altering Mo-MDSC inhibitory activity. Furthermore, scavenging of NO or iNOS knockdown prevented Mo-MDSC function, but did not affect PNT production or T cell suppression by G-MDSC. Taken together, our data indicates the independent suppressive pathways by which tumor-infiltrating MDSC-subpopulations impair T cell responses. These findings may enable the development of potential therapies to specifically block particular MDSC subpopulations in cancer and other diseases characterized by the accumulation of MDSC subsets and T cell dysfunction.

